# Current Status of Users of Postpartum Care Program at a Japanese Perinatal Center

**DOI:** 10.31662/jmaj.2023-0188

**Published:** 2024-06-03

**Authors:** Shunji Suzuki, Masako Eto

**Affiliations:** 1 Department of Obstetrics and Gynecology, Nippon Medical School, Tokyo, Japan; 2 Department of Obstetrics and Gynecology, Japanese Red Cross Katsushika Maternity Hospital, Tokyo, Japan

**Keywords:** Postpartum care program, mother, childcare exhaustion, mental health, Japan

In Japan, the Postpartum Care Program (PCP) has been established in 2014 to support healthy childcare for mothers and their families through the promotion of postpartum physical recovery and mental rest for mothers and children by fostering self-care skills in mothers themselves ^(1)^. The Guideline for PCP has been used as an implementation outline for postpartum care in each municipality since its publication in 2017 ^(1)^. Based on the guideline, the subsidy for the PCP has been provided to mothers such as (1) those who are anxious about childcare, (2) those who need consultation support and/or interaction support, and/or (3) those who need reduction or elimination of isolation based on the assessment by the person in charge in the municipality ^(1)^. According to the guideline ^(1)^, the postpartum care provided are as follows: (1) physical care, health guidance, and nutritional guidance for mothers, (2) psychological care for mothers, (3) care for appropriate breastfeeding, (4) specific guidance and consultation on child-rearing techniques, and (5) consultation and support for daily life. The PCP can be provided in three types ([a] short-term residential care [overnight or short stay], [b] day care, and [c] home visitational care [outreach]) and is provided at obstetric hospitals, obstetric private clinics, midwifery centers, and other institutes according to the standards set by the Ministry of Health, Labour and Welfare ^(1)^.

In our institute (one of the main perinatal centers in Tokyo, Japan), the PCP of short-term residential care has been used for 5 years ^(2)^. Therefore, to consider the current status of users of the PCP we examined the reasons to require the support by the PCP in the postpartum users in the past 2 years.

The study protocol was approved by the Ethics Committee of the Japanese Red Cross Katsushika Maternity Hospital.

This was a retrospective study of postpartum women within 4 months after delivery of live births at ≥22 weeks of gestation who were managed at our institute between January 2021 and October 2023. Overall, 209 women received the PCP during the study period. Of them, 174 with residency inside the district (a total of 174 visits) were examined for the study whereas the remaining ones were excluded because their residency was outside the district, making them ineligible for subsidies ^(1)^. We obtained the following information from their medical charts: nationality, age (≥35 years), parity, and reasons for and timing of receiving the PCP. In this study, 174 women who gave live births after the subject in the birth record list of our institute without receiving the PCP were compared on the same factors as control.

Data were expressed as number (%). Statistical analyses were conducted using SAS version 8.02 (SAS Institute, Cary, NC, USA). Cases and controls were compared using *X*^2^ or Fisher’s exact test for categorical variables, and *P* < 0.05 was considered to indicate statistical significance.

Table 1 presents the characteristics of the women who received the PCP and the reasons for receiving it. The percentages of women who were non-Japanese and nulliparous were higher in those who received the PCP than the control group (non-Japanese: 15% vs. 8%, *P* = 0.04; nulliparous: 71% vs. 48%, *P* < 0.01). Furthermore, the percentage of women aged ≥35 years was higher in those who received the PCP than the control group (45% vs. 32%, *P* = 0.01).

**Table 1.** Characteristics of Women Who Received or Did Not Receive the Postpartum Care Program and in This Study.

Postpartum Care Program use	Yes	No	*P*-value
Total number	174	174	
Nationality			
Japanese	148 (85)	160 (92)	
Non-Japanese	26 (15)	14 (8)	0.04
Maternal age			
35 years and above	79 (45)	56 (32)	0.01
Nulliparity	124 (71)	83 (48)	<0.01
Timing of use
<1 month after delivery	115 (66)	-	-
1-2 months after delivery	58 (33)	-	-
3-4 months after delivery	1 (1)	-	-
Reason for use*			
Rest (childcare exhaustion)	89 (51)	-	-
Acquisition of childcare techniques	67 (39)	-	-
Lack of support	19 (11)	-	-
Hospitalization of newborn	16 (9)	-	-
Anxiety about childcare	5 (3)	-	-
Depressive symptoms	4 (2)	-	-

Data are expressed as number (percentage, %). *There are duplications.

As shown in Table 1, 66% of the women received the PCP within 1 month after delivery. The most common reason for PCP use was childcare exhaustion (51%), whereas anxiety about childcare and depressive symptoms were observed in 3% and 2% of them, respectively.

The results of the current retrospective examination indicated that the use of PCP was associated with advanced maternal age, nulliparity, and foreign nationality. The risk of facing difficulties with child-rearing has been observed to be higher in mothers with advanced age than in young mothers ^(3), (4)^. Foreigners are more likely to have problems raising children isolated from the living area due to differences in lifestyle habits such as diet and language barriers ^(5)^. The results of this study are consistent with those of previous ones ^(3), (4), (5)^. In Japan, the number of elderly primiparous and foreign women is increasing year by year. Therefore, the number of women requiring the PCP is expected to continue increasing, although it cannot be denied that foreign women were the impact of the COVID-19 pandemic ^(6)^. Such an increase suggests that more and more mothers will complain of “childcare exhaustion” and wish to rest. Therefore, it is imperative to create a system that enables the mothers to take care of their children and get a good rest and that provides childcare support to mothers who are well rested so that they do not experience the same situation.

In this study, the most common complaint among women who needed the PCP was childcare exhaustion, whereas only a few percentage of women had symptoms of anxiety or depression. For example, in our recent study, the incidence of postpartum depression was 5.4% ^(7)^. Patients complicated by postpartum depressive symptoms sometimes may not eligible for the subsidies of the PCP because it will be covered by medical insurance for psychotherapy; therefore, the proportion of women with depressive and/or anxiety symptoms may not be high among the PCP users. In addition, the number of mothers with childcare exhaustion may increase due to the shift to nuclear families and the decline in communities in Japan.

Childcare exhaustion can occur to anyone and may have been previously taken lightly as a normal part of childcare; however, if this condition worsens, it may lead to postpartum depression ^(3), (8)^. As childcare exhaustion is naturally not covered by medical insurance, the PCP will be expected to play a pivotal role in preventing depression ^(1)^. Furthermore, to ensure that mothers with mental health problems can receive high-quality postpartum care anywhere in the country, it is necessary to eliminate regional disparities in subsidies ^(9)^. In the future, it is hoped that postpartum support will be expanded through multidisciplinary collaboration, which will help prevent postpartum depression and support raising healthy children ^(9)^. It is also necessary to build a system that allows for opportunities to consult with local administrative officials and/or psychiatrists without difficulty.

We understand the presence of serious limitations in this study aside from the small sample size. For example, only three factors were found to be associated with PCP use; many other important factors need to be evaluated such as education, marital status, employment, and family income ^(10)^. However, in this retrospective study, we could not examine these factors from the patients’ medical charts.

In June 2023, each municipality was notified that PCP should be made available for all women requiring support, not only those with mental health problems such as anxiety about childcare and depressive symptoms ^(10), (11)^. In other words, it is not the municipality but the woman who uses the PCP who decides whether she needs it. Therefore, the PCP will be a “universal service” in Japan and will be a large step toward the nationwide development of a full range of the PCP. Furthermore, the number of women who are hesitant to use the PCP due to concerns about the screening process by the municipality is expected to decrease.

Finally, in this study, we evaluated only women with residency inside the district because those with residency outside the district are sometimes ineligible for the subsidies ^(1)^. We believe that these subsidy restrictions are another issue that needs to be addressed.

Based on the results of the present study, the number of mothers requiring the PCP will continue to increase in the future. Thus, further expansion of the PCP will be needed.

## Article Information

### Conflicts of Interest

None

### Sources of Funding

This article was conducted as part of the Ministry of Health, Labour and Welfare’s Comprehensive Research Project for the Development of Healthy Next Generations.

### Author Contributions

Shunji Suzuki: project development, data management, data analysis, and manuscript writing/editing.

Masako Eto: project development, data collection, data analysis, and manuscript writing/editing.

### Informed Consent

Patients’ informed consent for publication of this report was obtained.

### Approval by Institutional Review Board (IRB)

The study protocol was approved by the Ethics Committee of the Japanese Red Cross Katsushika Maternity Hospital (K2023-16).
